# Another Crusade

**Published:** 1904-07

**Authors:** 


### ANOTHER CRUSADE.

This is an age of crusades! We have had all kinds of them—and our profession has not escaped! But here at least is one which all of us can endorse—the anti-patent-medicine crusade. An article appeared in the May number of the *Ladies' Home Journal* which is likely to bring home to a great many people the enormity of this gigantic evil, which is doing so much to undermine the health and, perhaps, we may add, the morals of a large proportion of our people. Editor Bok points out a fact which is known to every physician, that many of these so-called "patents," are nothing but alcoholic beverages masquerading as "tonics," "bitters," "catarrh and dyspepsia cures," etc. The article is a brave one in that it lays its publishers open to suit—since it is by no means easy to prove to the satisfaction of a court that statements regarding alcoholic strength are absolutely correct. There are, unfortunately, too many chances for error in the work of the analytical chemist. The publishers of the *Journal* have already had a suit for $200,000 damages entered against them by R. V. Pierce for statements made in this article regarding the "Favorite Prescription." They were misled in reposing confidence in the accuracy of the Hagar formula for this well-known nostrum.

This is a brave fight and deserves the hearty support of the medical profession. The good done by such an article, published in a journal which goes monthly into more than a million homes, can not be overestimated. And happily this is not the only journal to take this stand. The advertising pages of the magazines of the better class are being rapidly purged of the whole dirty brood of quack-doctor and patent-medicine "ads." For instance, *Everybody's Magazine* no longer receives them, nor does the *Review of Reviews.* Some time ago we recorded the fact that the Methodist periodicals had come over to the righteous minority and were setting a good example to the horde of other religious journals which still persist in sin. We shall hope for a "change of heart" that will soon place the influence of the rest of these influential religious organs on the side of decency.

Yes, this is a crusade that we can all enter into heart and soul. The alcoholic patents, however, are not the only dangerous ones. There are some which contain such dangerous drugs as morphin and cocain; others which are full of bromides, digitalis, and other potent poisons; there are hundreds of them which consist mainly of coal-tar remedies and other heart depressants. It is probably too much to hope that the sale of these nostrums can be suppressed —but certainly the people who buy them ought to know something of what they are taking. It is a strange thing but a great many people who would not think of "tinkering" with a broken watch will not hesitate to tamper with that most delicate of all mechanisms—the human body. Almost every man has a specific for at least one of the many ills with which humanity is afflicted; and unfortunately any man with the "nerve" and a little money can unload the product of his credulity and ignorance upon a longsuffering people. To became a physician, or even to sell "salts" and wall-paper in a country drug-store, it is now necessary to pass a trying examination before an inquisitive State board; but any parish priest, blacksmith, or Digger Indian may "invent" a new concoction of impossibles and foist it on a confiding community as a "wonderful discovery."—*Medical Standard.*

